# Preparation and Characterization of di- and Tricarboxylic Acids-Modified Arabinogalactan Plasticized Composite Films

**DOI:** 10.3390/polym15091999

**Published:** 2023-04-23

**Authors:** Yuriy N. Malyar, Valentina S. Borovkova, Alexander S. Kazachenko, Olga Yu. Fetisova, Andrey M. Skripnikov, Valentin V. Sychev, Oxana P. Taran

**Affiliations:** 1 Institute of Chemistry and Chemical Technology, Krasnoyarsk Science Center, Siberian Branch, Russian Academy of Sciences, Akademgorodok 50/24, Krasnoyarsk 660036, Russia; 2 School of Non-Ferrous Metals and Materials Science, Siberian Federal University, pr. Svobodny 79, Krasnoyarsk 660041, Russia

**Keywords:** polysaccharides, arabinogalactan, biofilms, carboxylic acids, cross-linking, methylene blue

## Abstract

To ensure the high quality of water, it is necessary to remove toxic pollutants. At present, purification of water is implemented using various sorbents. The efficient sorption materials are modified polysaccharides. In this study, we report on a new environmentally friendly method for modifying larch hemicellulose—arabinogalactan (AG)—with polybasic carboxylic acids (citric, succinic, oxalic, and adipic) to obtain composite materials. The synthesized AG derivatives have been explored by a complex of physicochemical methods, including gel permeation chromatography (GPC), Fourier-transform infrared spectroscopy (FTIR), thermogravimetric analysis (TGA), X-ray diffractometry (XRD), scanning electron microscopy (SEM), and sorption capacity investigations. It is shown that the heat treatment results in the formation of additional inter- and intramolecular bonds between carboxylic acids and polysaccharide molecules. The formation of ester bonds has been confirmed by the appearance of absorption bands in the IR spectra in the range of 1750–1690 cm^−1^. It has been found, using the TGA study, that the most thermally stable (up to 190 °C) sample is arabinogalactan oxalate obtained under heat treatment. The SEM study of the synthesized AG films has shown that the modified samples have the homogeneous film surface ensured by cross-linking. It has been established, when studying the sorption properties of the AG derivatives, that AG succinate (82.52%) obtained by lyophilization has the highest sorption capacity, due to the developed mesoporous surface, which, in turn, makes the synthesized films promising eco-friendly materials for use as drug carriers, sorbents, and water treatment agents.

## 1. Introduction

Industrial, agricultural, medical, and household pollution have become the main threats to the environment and humanity [1]. Heavy metals, dyes, and pesticides are among the most persistent hazardous pollutants, in particular, for surface and groundwater. Pollutants are removed from aquatic environments by various physical, chemical, and physicochemical methods [2,3], for example, electrochemical destruction, ozonation, ion exchange reactions, activated carbon and silica gel sorption, membrane filtration, and others [3,4,5]. Among the available water purification techniques, the use of sorption materials has proven to be the most efficient, due to their low cost, functionality, and possibility of further extraction and reuse [4]. Sorption substances can be organic, mineral, or biological, which makes them preferred as completely safe for the environment [6] and not inferior to the conventional sorbents in terms of performance [5]. Particular interest is focused on natural polymers [2], which are good candidates, both fundamentally and economically [7], for purification of wastewater and soil from metal ions and other pollutants [8].

Biopolymers, especially cellulose derivatives [9,10], natural gums [5,11], collagen [9], chitosan [9,12], cyclodextrins [13], and others, have already proven themselves as environmentally friendly sorbents. The properties of each of these sorbents can be different, depending on the porosity of their structure, specific surface area, surface chemistry, and structural characteristics. In some cases, it is reasonable to modify polymeric materials to improve the chemical resistance and productivity. One of the simplest and most efficient modification techniques is based on esterification reactions, with the use of polyfunctional compounds, e.g., polybasic carboxylic acids. The latter belong to the cross-linkers, that are being intensively studied due to their nontoxicity, low cost, and ease of processing upon cross-linking [14]. Such polyfunctional compounds form covalent bonds with polymer chains and create spatially cross-linked structures, which improves their mechanical properties, stability, and moisture resistance. Previously, this modification method was applied to commercial high-molecular-weight polysaccharides to obtain starch films [14], gum-based hydrogels [15], cellulose, and cellulose derivatives [16]. However, other polysaccharides, for example, hemicelluloses, being the second most abundant renewable natural polymers [17], have not been studied in this direction. 

Arabinogalactan is a hemicellulose that forms the basis of angiosperm gums, especially of the genus Larix [18]. Arabinogalactan II [18,19], present in large amounts in larch wood [20], and widespread in the boreal forests of Siberia and North America, is known to be the most commercially available. Arabinogalactan macromolecules consist of a β-1,3-galactan main chain and β-1,6-galactan side chains, with attached α-1-arabinofuranosyl and β-1-arabinopyranosyl residues [18,19]. These polysaccharides exhibit a complex of valuable properties [21,22,23], the most important of which are their water solubility, high biological activity, low toxicity, ease of processing, low cost, and biodegradability, which undoubtedly make them an excellent basis for synthesis of films, coatings, and sorption materials. 

In this study, the nontoxic, inexpensive, and available carboxylic acids (oxalic, succinic, citric, and adipic) were chosen for modifying arabinogalactan. The selected acids had to be fundamentally different in acidity, water solubility, and molecular structure length in order to demonstrate the role of internal molecular differences between cross-linkers. Accordingly, it is assumed that the modification of arabinogalactan with polyfunctional carboxylic acids will ensure the formation of alternative biomaterials, including sorbents, which can easily be decomposed and processed by microorganisms (bacteria, fungi, and algae). 

The aim of this study was to synthesize and explore new composites based on Siberian larch wood arabinogalactan (*Larix sibirica Ledeb.*) modified with polyfunctional carboxylic acids. The results obtained provide the information required for the development of biodegradable sorption materials, which will contribute to wider use of natural polymers and wood pulp. 

## 2. Materials and Methods

### 2.1. Reagents 

In the synthesis, larch wood (*Larix Sibirica*) arabinogalactan (Fibrolar C, Wood Chemistry LLC, Irkutsk, Russia), sodium hydroxide (Ekos-1, Moscow, Russia), ethyl alcohol (Konstanta-farm M, Moscow, Russia), citric acid (Sigma, Saint Petersburg, Russia), succinic acid (CHRS, Ufa, Russia), oxalic acid (CHRS, Ufa, Russia), and adipic acid (CHRS, Ufa, Russia) were used. 

### 2.2. Synthesis of the Arabinogalactan Derivatives with the Polybasic Carboxylic Acids

The arabinogalactan derivatives with polybasic carboxylic acids were obtained using modified procedures [24,25].

Arabinogalactan was modified with carboxylic acids: citric, succinic, oxalic, and adipic. Five grams of acid was dissolved in 50 mL of distilled water under stirring. After the complete dissolution, 5 g of arabinogalactan was added. To prevent hydrolysis of arabinogalactan in an acidic medium, the reaction mixture pH was adjusted to 3.5 and 1 N NaOH was added. The obtained solutions were then stirred for 24 h.

Each resulting reaction mixture was divided into two equal portions. The first portion (25 mL) was placed in an oven and dried at 50 °C for 24 h, after which the temperature in the oven was raised to 80 °C and kept at this level for 3 h.

The second portion was frozen at −20 °C and the sample was freeze-dried for 24 h.

The synthesized derivatives were ground in a porcelain mortar, put in a funnel, and washed with ethyl alcohol to remove unreacted carboxylic acids, until the wash water showed a neutral pH value. As a result, arabinogalactan citrates (AG/CA), succinates (AG/SA), oxalates (AG/OA), and adipates (AG/AA) were obtained.

**Figure 1:**
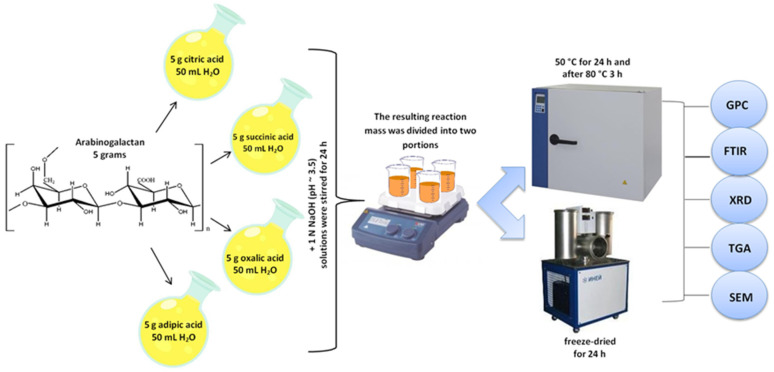
Scheme of the process to obtain the arabinogalactan derivatives.

The process is schematically illustrated in Figure 1.

### 2.3. Physicochemical Study

#### 2.3.1. Gel Permeation Chromatography

The molecular weight characteristics (weight average and number average molecular weights, *M*_w_ and *M*_n_, respectively) and the polydispersity index (PDI)) of arabinogalactans modified with carboxylic acids were determined by gel permeation chromatography on an Agilent 1260 Infinity II Multi-Detector GPC/SEC System (Agilent Technologies, Santa Clara, CA, USA) with triple detection: a refractometer (RI), a viscometer (VS), and light scattering (LS). The separation was performed on two combined PL Aquagel-OH Mixed-M columns, using 0.1 M NaNO_3_ and 250 ppm NaN_3_ in water as a mobile phase. The columns were calibrated using polyethylene glycol standards (Agilent, Santa Clara, CA, USA). The eluent flow rate was 1 mL/min and the sample volume was 100 μL. Before the analysis, the samples were dissolved in the mobile phase (5 mg/mL) and filtered through a 0.45 µm Agilent PES membrane filter (Merck Millipore, Burlington, MA, USA). The data collection and processing was performed using the Agilent GPC/SEC MDS software version 2.2.

The molecular weights, *M*_w_ and *M*_n_, and the PDI value were determined from the calibration curve obtained using polyethylene glycol polydisperse standards.

#### 2.3.2. Fourier-Transform Infrared Spectroscopy

The IR spectra in the range 4000–500 cm^−1^ were recorded on a Tensor-27 IR Fourier spectrometer (Bruker Optik Gmbh, Ettingen, Germany) at the Krasnoyarsk Regional Center for Collective Use, Siberian Branch of the Russian Academy of Sciences. The spectral data were processed using the OPUS software package, version 5.5. Samples for the IR spectroscopy investigations were pressed into tablets in a potassium bromide matrix. The sample preparation conditions (time of mixing with potassium bromide, pressure, and evacuation time) were invariable. The substance concentration was constant: 4 mg of the substance per 1000 mg of KBr.

#### 2.3.3. X-ray Diffraction

The X-ray diffraction study was carried out on a DRON-3 X-ray diffractometer (Burevestnik, St. Petersburg, Russia) (monochromatic CuKα radiation, λ = 0.154 nm) at a voltage of 30 kV and a current of 25 mA. The scanning step was 0.02 deg and the intervals were 1 s per data point. The measurements were performed in the Bragg angle (2Θ) range from 5.00 to 70.00.

#### 2.3.4. Scanning Electron Microscopy

The surface morphology of the arabinogalactan film samples was studied using a scanning electron microscope, Hitachi TM-4000 (Hitachi High-Tech Corporation, Tokyo, Japan), at an accelerating voltage of 15 kV and a magnification from ×100 to ×10,000, with a resolution of 30 nm. The SEM images were processed using the ImageJ software, version 1.8.0_112. 

#### 2.3.5. Thermogravimetry Analysis

The thermogravimetry study was carried out on a NETZSCH TG 209 F1 thermal analyzer (NETZSCH STA 449 F1 Jupiter instrument, Netzsch, Selb, Germany) and the data obtained were analyzed. The thermal decomposition of the samples was analyzed in nitrogen in the temperature range from 25 to 700 °C. The protective and blowout gas flow rates were 20 mL/min. The samples were heated in cylindrical corundum crucibles in a dynamic temperature regime (10 °C/min). A TG 209 F1 analyzer was calibrated using the instructions and reference manifestations that appear with the instrument and weighing the samples on an XFR-125E laboratory balance. The measurement data were processed in the NETZSCH Proteus Thermal Analysis 4.8.4 software supplied with the instrument.

#### 2.3.6. Sorption Capacity Analysis 

A mass of 0.1 g of each of eight samples was added to 10 mL of methylene blue, with a concentration of 0.00625 g/L. The solutions with the adsorbent were mixed in a shaker for 3 h at a speed of 160 rpm at room temperature (~25 °C).

After mixing, the samples were filtered through an Agilent PES polytetrafluoroethylene (PTFE) membrane syringe filter (Merck Millipore, Burlington, MA, US), with a diameter of 25 mm and a pore size of 0.45 μm.

The optical density of the samples was determined on a SPEKOL-1300 spectrophotometer (Analytik Jena AG, Germany) using k8-24.10.A photometric cuvettes 24 × 16 × 40 mm in size, with a wall thickness of 3 mm and an optical path of 10 mm.

For all the samples, distilled water was used as a control solution. The measurements were carried out at a methylene blue absorption length of 665 nm [26].

The methylene blue concentration in the solution after sorption was determined as

(1) $$C=\frac{A}{A_{0}}\times C_{0}$$

where *C*_0_ and *C* are the methylene blue concentrations and *A* and *A*_0_ are the optical density of the methylene blue solutions before and after the adsorption, respectively.

The sorption efficiency *H* (%) of the film materials obtained was calculated as [27]:

(2) $$H\;\left(\%\right)=\frac{\left(C_{0}-C\right)}{C_{0}}\times 100\%.$$

## 3. Results

The cross-linking yielded eight modified arabinogalactan samples, the structures and properties of which were studied by physicochemical methods.

### 3.1. Gel Permeation Chromatography

#### 3.1.1. Thermal Drying of the Arabinogalactan Films

Among the arabinogalactan samples obtained using the thermal drying, AG/AA and AG/OA are completely soluble in aqueous media and AG/SA exhibits partial solubility. AG/CA is insoluble in aqueous media, which is undoubtedly related to the structure of a cross-linking agent containing three carboxyl groups tightly bound to the arabinogalactan OH groups [28]. With prolonged heating, the carboxyl groups of citric acid are converted into anhydrides, which react with polysaccharides’ hydroxyls, forming cross-links [29]. Similar results have been achieved with other polysaccharides cross-linked with citric acid [28,29,30]. 

The water-soluble arabinogalactan samples were studied by GPC. The molecular weight characteristics obtained are given in Table 1.

**Figure 2:**
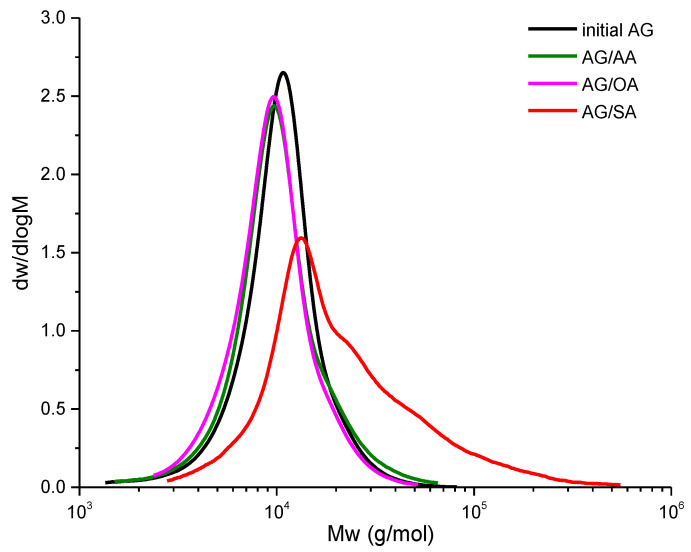
Molecular weight distributions in the arabinogalactan samples (thermal drying).

According to the data obtained, the molecular weights of the AG adipate and oxalate samples are somewhat lower than that of the initial arabinogalactan. This may be caused by the two competing processes: esterification of hydroxyl groups and hydrolysis of the glycosidic bonds. In the case of using oxalic acid, which is fairly strong, partial hydrolysis of the arabinogalactan side chains occurs, which is reflected in the largest shift of the molecular weight distribution (MWD) peak to the low-molecular-weight region (Figure 2). 

The absence of cross-linking can be explained by the steric effect, by which the access to the free carboxyl group is complicated by the small size of the molecule and the need for a close approach of an additional arabinogalactan molecule in the cross-linking reaction. In the case of using adipic acid, the MWD also shifts, but, due to the low pKa value, the reactivity of the carboxyl group is weak, which complicates the esterification reaction.

#### 3.1.2. Freeze-Drying of the Arabinogalactan Films

According to the data in Table 2, the molecular weight of the sublimated AG derivatives is also lower than that of the initial arabinogalactan. However, this is due to the fact that during freeze-drying, the solvent and carboxylic acid sublimate, therefore, the final cross-linking reaction does not occur [31], i.e., the esterification reaction is with only one carboxylic acid group or this does not occur at all.

**Figure 3:**
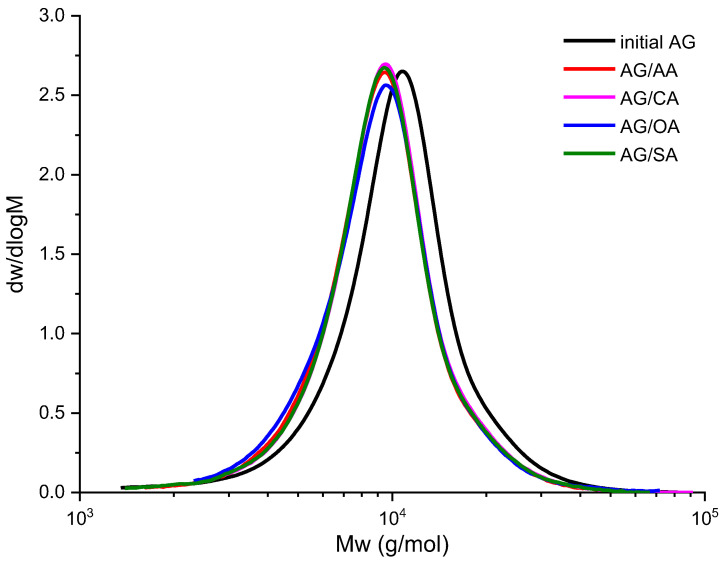
Molecular weight distributions in the arabinogalactan samples (freeze-drying).

The MWDs (Figure 3) of the arabinogalactan samples modified with all the investigated carboxylic acids shift toward lower molecular weights, which demonstrates the predominance of hydrolysis of side chains and the esterification reactions with only one carboxylic acid group (Section 3.2).

### 3.2. Fourier-Transform Infrared Spectroscopy of the Arabinogalactan Films

The IR spectrum of the initial arabinogalactan [32] includes high-intensity absorption bands characteristic of bending vibrations of cycles (716, 781, 884, 1085, and 1162 cm^−1^). In the IR spectrum, the carbonyl group exhibits a very strong absorption around 1647 cm^−1^. The absorption bands characteristic of the C–O stretching vibrations appear in the regions of 1085 and 1162 cm^−1^. The vibrations of the hydroxyl groups are observed around 2913 cm^−1^. The broad peak characteristic of the associated hydroxyl groups can be seen at 3385 cm^−1^.

**Figure 4:**
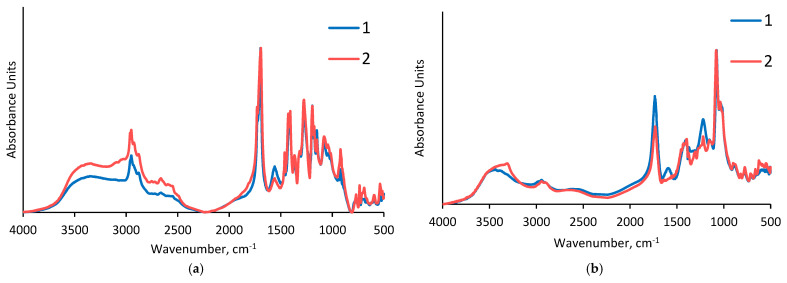

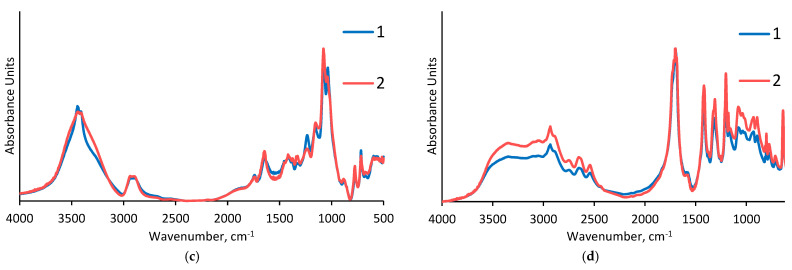
FTIR spectra of the AG samples: (**a**) AG/AA, (**b**) AG/CA, (**c**) AG/OA, and (**d**) AG/SA under: (**1**) thermal drying and (**2**) freeze-drying.

The effect of the method of drying the arabinogalactan derivatives with the polybasic carboxylic acids on the spectral characteristics is illustrated in Figure 4.

According to the data presented in Figure 4, the samples of the arabinogalactan derivatives with polybasic carboxylic acids obtained by different methods yield similar absorption bands and their spectra only differ in intensity. In the middle portions (1750–1690 cm^−1^) of the spectra, the absorption bands of esters of carboxylic acids appear. The maximum absorption intensities of the modified arabinogalactans correspond to the bands at 1699, 1701, and 1724 cm^−1^, which are related to the C=O stretching vibrations [14]. However, the absorption bands of esters in arabinogalactan films cross-linked by oxalic acid appear with the lowest intensity (Figure 4c).This phenomenon is associated with the relatively high dissociation constants of oxalic acid, as a result of which, together with esterification, the hydrolysis of arabinogalactan side chains occurs (Figure 2 and Figure 3).

### 3.3. X-ray Diffraction Analysis of the Arabinogalactan Films

**Figure 5:**
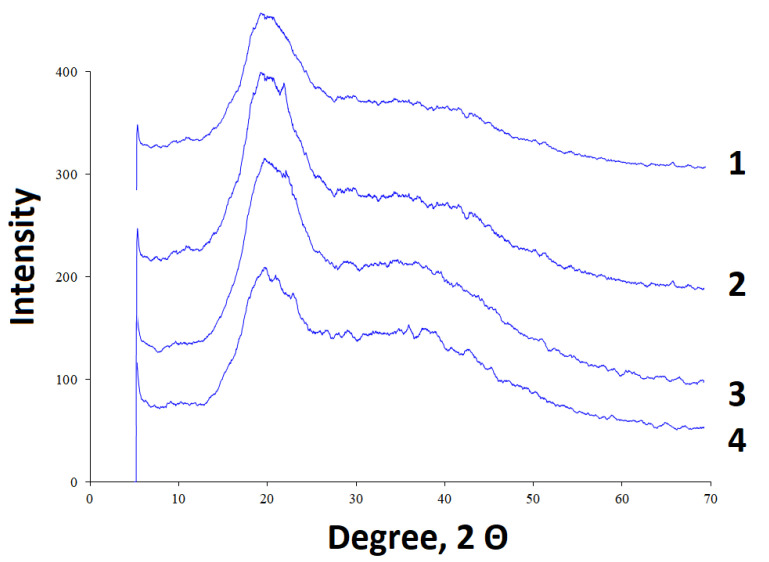
X-ray diffraction patterns of the (**1**) AG/AA, (**2**) AG/CA, (**3**) AG/OA, and (**4**) AG/SA samples obtained by thermal drying.

The arabinogalactan derivatives with polybasic carboxylic acids were analyzed by X-ray diffraction (Figure 5).

According to the data reported in [22,23], the initial arabinogalactan is an X-ray amorphous substance. The X-ray diffraction patterns of arabinogalactan contain a halo in the range from 15 to 25° 2Θ. During esterification of arabinogalactan with polybasic carboxylic acids, the amorphization is intensified, which manifests itself in a weakening of the peaks in the range from 15 to 25° 2Θ. According to the data presented in Figure 5, the weakest amorphization is observed in AG/CA and the strongest one, in the AG/AA sample. It is noteworthy, that the amorphization of the target product following esterification with polybasic acids is much weaker than following modification of arabinogalactan with sulfate groups [22], which is probably due to the absence of depolymerization reactions during the esterification.

### 3.4. Scanning Electron Microscopy of the Arabinogalactan Films

The SEM method was used to characterize the morphology of arabinogalactan films. 

**Figure 6:**
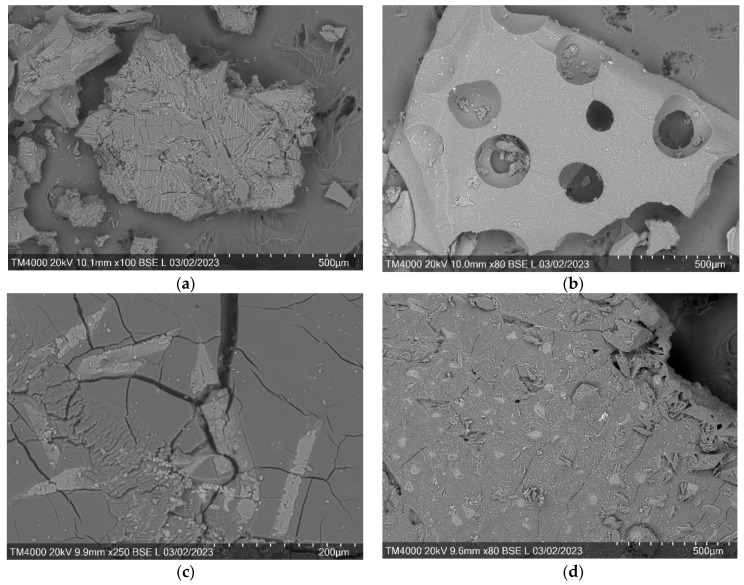
SEM scans of arabinogalactan and its derivatives (thermal drying): (**a**) AG/AA, (**b**) AG/CA, (**c**) AG/OA, and (**d**) AG/SA.

According to the SEM data (Figure 6), the morphology of AG particles changes significantly after modification. If the original AG consists of aggregated and single particles of various shapes and sizes [20,33], then in the case of modified samples obtained under heat treatment, the surface of the films is more uniform and homogeneous, but with some defects, similar to starch films [14]. This phenomenon is explained by the smooth removal of the solvent from the system, which makes it possible to obtain a sufficiently dense material. Gebresas G.A. et al. [34] have already described a similar morphology of films of polysaccharides cross-linked with succinic acid, where a relatively smooth granular structure is observed. 

**Figure 7:**
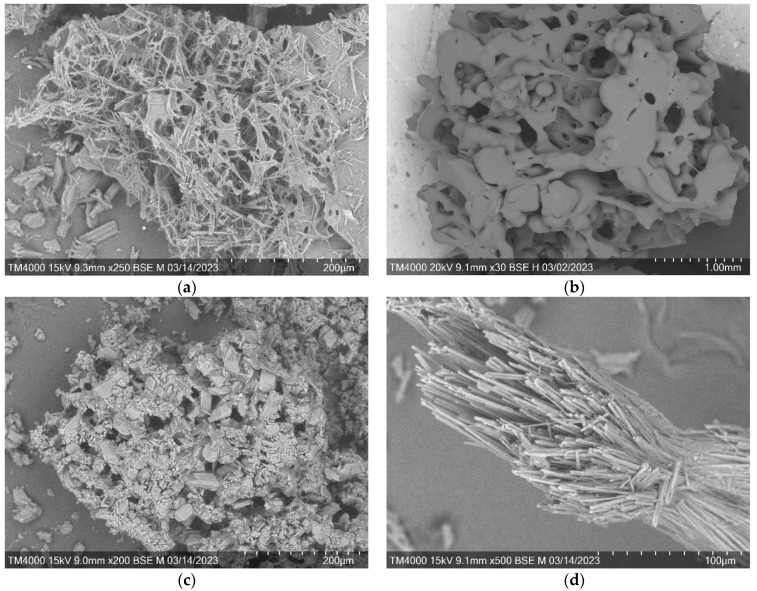
SEM scans of arabinogalactan and its derivatives (freeze-drying): (**a**) AG/AA, (**b**) AG/CA, (**c**) AG/OA, and (**d**) AG/SA.

As for the samples obtained by sublimation of the solvent, the output materials are reminiscent of a certain frame with many elongated channels (Figure 7). Similar phenomena have been demonstrated with glucuronoxylan in the work by Hussain M.A. et al. [29]. 

However, a sample of arabinogalactan cross-linked with succinic acid acquires a fibrous structure after freezing (Figure 7d). It is assumed that, thanks to these fibers, this sample will be able to manifest itself as a sorbent material [35].

### 3.5. Thermogravimetry Analysis of the Arabinogalactan Films

It is well known that the shape of thermogravimetry (TG) curves is determined by the heterogeneous nature of thermal decomposition, occurrence of various chemical reactions, destruction of hydrogen bonds, and conformational and phase transitions [36]. Under thermal decomposition, the thermal destruction of arabinogalactan and its derivatives will apparently proceed, which is accompanied by the destruction of the glycosidic bonds. Moreover, thermal stability is an important characteristic of arabinogalactan, which largely determines its application potential.

**Figure 8:**
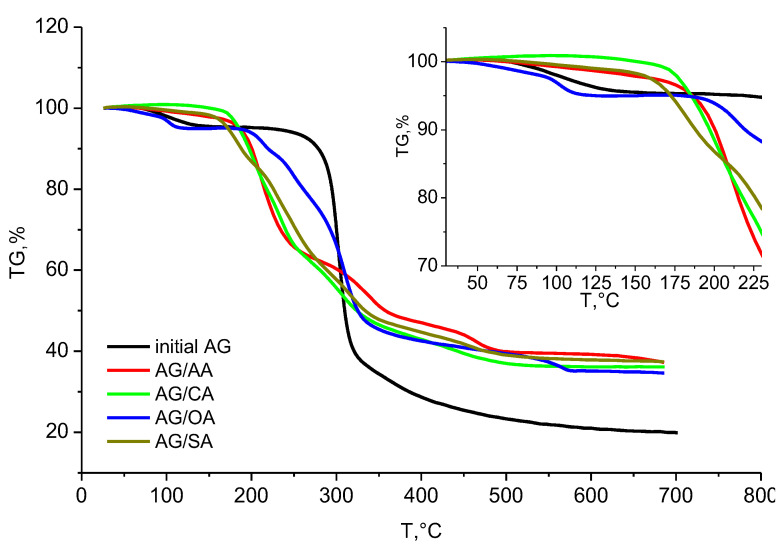
TG curves of arabinogalactan and its derivatives (thermal drying) that passed through the heat treatment.

Figure 8 graphically interprets the TGA data on arabinogalactan and its thermally dried derivatives. The temperature intervals, and the corresponding weight losses after heating, of the investigated samples are listed in Table 3.

The thermal decomposition of arabinogalactan in the temperature range 30 to 700 °C occurred in three stages. At the first stage, already at a temperature of 95 °C, arabinogalactan started losing crystallization moisture and, then, sorbed moisture. By 130 °C, the weight loss was 4.1 wt.%. A further increase in temperature to 230 °C barely affected the arabinogalactan structure (the weight loss in the range 130–230 °C was 1.19 wt.%). This fact can be explained by the difficulty of breaking hydrogen bonds between water molecules and polar functional groups of polysaccharides [37]. 

The main decomposition of arabinogalactan occurred at the second thermolysis stage, in the temperature range 230 to 500 °C. 

At this stage, the maximum sample weight loss (71.49 wt.%) was observed. It is caused mainly by destruction of the glycosidic bonds and the aromatization and depolymerization of the arabinogalactan monosaccharide components [38]. The third stage (500–700 °C) was characterized by a minor weight loss (3.33 wt.%), which may be due to the aromatization of the arabinogalactan structure leading to the formation of a coke residue [39]. At the end of pyrolysis (700 °C), the residue was 19.89%. 

The modification of arabinogalactan with carboxylic acids changes significantly the nature of its thermal decomposition (Figure 8). After thermal drying, the temperature of the onset of the main decomposition in the modified samples decreased by 70–80 °C (Table 3), except for sample AG/OA_thermal_, which remained heat resistant up to 190 °C. The range of the stage of the main decomposition of the investigated samples noticeably broadened, which is indicative of a significant modification of the arabinogalactan structure by carboxylic acids. Samples AG/AA_thermal_, AG/CA_thermal_, and AG/SA_thermal_ completed their thermal decomposition by 500 °C, while the weight losses in the samples at this thermal degradation stage were 57.64%, 62.64%, and 59.22%, respectively. The interval of the main thermal destruction of sample AG/OA_thermal_ was much wider: from 190 to 571 °C (the weight loss was 59.28%). In addition, by the end of pyrolysis, sample AG/OA_thermal_ had a smaller residue, which may suggest a deeper transformation of its structure with the formation of the least thermally stable fragments.

Generally, the drying method used does not affect the thermal behavior of the samples. Nevertheless, the arabinogalactan adipate, citrate, and oxalate samples passed through lyophilization undergo stronger thermal degradation as compared with the thermally dried samples. This is proven by the smaller weight loss at the main decomposition stage and the smaller coke residue for samples AG/AA_freeze_, AG/CA_freeze_, and AG/OA_freeze_, as compared with samples AG/AA_thermal_, AG/CA_thermal_, and AG/OA_thermal_ (Table 3).

At the same time, the method for drying arabinogalactan succinate does not affect its thermal stability. Despite the fact that, during the main destruction, sample AG/SA_thermal_ decomposes to a greater extent than sample AG/SA_freeze_, the coke residues in these samples are approximately the same. 

### 3.6. Sorption Capacity of the Arabinogalactan Films

The possibility of using the synthesized materials in water treatment depends on their ability to absorb pollutants. In determining the sorption properties, marker substances are used, which simulate various classes of pollutants; for example, methylene blue (MB) simulates toxins with a weight of up to 500 Da. Table 4 lists the data on the sorption capacity of the modified arabinogalactan samples under study. 

The data on sorption of MB by the investigated substances were obtained. It was shown that sample AG/SA_freeze_ has the highest sorption capacity, which amounted to 82.52% of methylene blue from the solution after 3 h. Similar values (67.49–70.64%) were obtained for the sorption capacity of the samples passed through the thermal drying with adipic, citric, and succinic acids. The sorption activity of the obtained samples can be explained by the nature of methylene blue, which is a salt with a high degree of dissociation, which contributes to its binding on the surface of modified arabinogalactan [40]. The increase in the absorption capacity of the samples obtained by freeze-drying is explained by the developed mesoporous surface (Figure 7), because the methylene blue molecule size is larger than 2 nm [41].

The modification with oxalic acid during drying, both lyophilic and thermal, does not significantly increase the sorption capacity, due to the predominance of hydrolysis of side chains over the esterification and cross-linking. 

## 4. Conclusions

In this work, a method for modifying arabinogalactan using thermal and freezing treatment has been developed. The formation of inter-/intra-molecular bonds was confirmed by the changes in the molecular weight distribution, the signals of ester bonds in the FTIR spectra, and the changes in the nature of surfaces according to SEM data. The study of the sorption properties of arabinogalactan derivatives using methylene blue showed that the removal of impurities from aqueous media is achieved due to covalent bonding with carboxyl groups on the surface and the developed mesoporous structure of samples obtained in freeze-drying. Thus, the obtained new derivatives of arabinogalactan with polybasic carboxylic acids can be considered as promising materials, with the properties of sorbents, films, and coatings.

## Figures and Tables

**Table 1:** Molecular weight characteristics of the initial arabinogalactan and its derivatives.

Sample	*M_n_*, g/mol	*M_w_*, g/mol	PDI
Initial AG	9101	11,580	1.27
AG/AA	8758	11,619	1.33
AG/CA	‒	‒	‒
AG/OA	8484	10,567	1.25
AG/SA	15,344	33,346	2.17

**Table 2:** Molecular weight characteristics of the initial arabinogalactan and its derivatives.

Samples	*M*_n_, g/mol	*M*_w_, g/mol	PDI
Initial AG	9101	11,580	1.27
AG/AA	8219	10,138	1.23
AG/CA	8547	10,381	1.22
AG/OA	8279	10,266	1.24
AG/SA	8209	10,222	1.25

**Table 3:** Stages of thermal decomposition of arabinogalactan and its derivatives.

Sample	Drying Method	$\frac{\mathrm{Temperature}\;\mathrm{Range},{}^{\circ}C}{\mathrm{Weight}\;\mathrm{Loss},\%}$			Residue at 700 °C, %
AG	‒	$\frac{30–230}{5.29}$	$\frac{230–500}{71.49}$	$\frac{500–700}{3.33}$	19.89
AG/AA	Thermal drying	$\frac{30–161}{2.47}$	$\frac{161–500}{57.64}$	$\frac{500–700}{2.63}$	37.26
AG/CA		$\frac{30–161}{0.39}$	$\frac{161–500}{62.64}$	$\frac{500–700}{0.85}$	36.12
AG/OA		$\frac{30–190}{5.32}$	$\frac{190–571}{59.28}$	$\frac{571–700}{0.80}$	34.60
AG/SA		$\frac{30–150}{1.72}$	$\frac{150–500}{59.22}$	$\frac{500–700}{1.68}$	37.38
AG/AA	Freeze-drying	$\frac{30–161}{1.78}$	$\frac{161–500}{61.84}$	$\frac{500–700}{2.24}$	34.14
AG/CA		$\frac{30–170}{1.21}$	$\frac{170–500}{63.15}$	$\frac{500–700}{2.08}$	33.56
AG/OA		$\frac{30–190}{5.12}$	$\frac{190–571}{61.48}$	$\frac{571–700}{1.11}$	32.29
AG/SA		$\frac{30–150}{1.95}$	$\frac{150–500}{58.94}$	$\frac{500–700}{1.43}$	37.68

**Table 4:** MB adsorption performance of AG-based composite films.

Sample		Concentration, g/L	Sorption Efficiency (H), %
Control		0.00625	‒
AG/AA	Thermal drying	0.003307	70.64
AG/CA		0.003438	67.49
AG/OA		0.005099	27.63
AG/SA		0.003399	68.43
AG/AA	Freeze-drying	0.004028	53.33
AG/CA		0.004805	34.68
AG/OA		0.004380	44.88
AG/SA		0.002812	82.52

## Data Availability

All data generated during this study are included in the article.
